# *miR-669a-5p* promotes adipogenic differentiation and induces browning in preadipocytes

**DOI:** 10.1080/21623945.2022.2030570

**Published:** 2022-01-30

**Authors:** Xiaoqiong Tan, Tingting Zhu, Linqiang Zhang, Lin Fu, Ying Hu, Huiqin Li, Chengbin Li, Jingjing Zhang, Bin Liang, Jing Liu

**Affiliations:** a Key Laboratory of Animal Models and Human Disease Mechanisms of the Chinese Academy of Sciences & Yunnan Province, Kunming Institute of Zoology, Chinese Academy of Sciences, Kunming, China; b Kunming College of Life Science, University of Chinese Academy of Sciences, Kunming, China; c Department of Respiratory and Critical Care Medicine, The First People’s Hospital of Yunnan Province, Kunming, China; d Department of Respiratory and Critical Care Medicine, The Affiliated Hospital of Kunming University of Science and Technology, Kunming, China; e Center for Life Sciences, School of Life Sciences, Yunnan University, Kunming, China; f School of Traditional Chinese Medicine, Yunnan University of Chinese Medicine, Kunming, China

**Keywords:** 3T3-L1, C3H10T1/2, miR-669a-5p, adipocyte browning, adipocyte differentiation, microRNA

## Abstract

Obesity is a major global health issue that contributes to the occurrence of metabolic disorders. Based on this fact, understanding the underlying mechanisms and to uncover promising therapeutic approaches for obesity have attracted intense investigation. Brown adipose tissue (BAT) can help burns excess calories. Therefore, promoting White adipose tissue (WAT) browning and BAT activation is an attractive strategy for obesity treatment. MicroRNAs (miRNAs) are small, non-coding RNAs, which are involved in regulation of adipogenic processes and metabolic functions. Evidence is accumulating that miRNAs are important regulators for both brown adipocyte differentiation and white adipocyte browning. Here we report that the expression of *miR-669a-5p* increases during the adipogenic differentiation of 3T3-L1 and C3H10T1/2 adipocytes. *miR-669a-5p* supplementation promotes adipogenic differentiation and causes browning of 3T3-L1 and C3H10T1/2 cells. Moreover, the expression of *miR-669a-5p* is upregulated in iWAT of mice exposed to cold. These data demonstrate that *miR-669a-5p* plays a role in regulating adipocyte differentiation and fat browning.

**Abbreviations**: *Acadl*: long-chain acyl-Coenzyme A dehydrogenase; *Acadm*: medium-chain acyl-Coenzyme A dehydrogenase; *Acadvl*: very long-chain acyl-Coenzyme A dehydrogenase, very long chain; *Aco2*: mitochondrial  aconitase 2; BAT: brown adipose tissue; *Bmper*: BMP-binding endothelial regulator; *Cpt1-b*:carnitine palmitoyltransferase 1b; *Cpt2*: carnitine palmitoyltransferase 2; *Crat*: carnitine acetyltransferase; *Cs*: citrate synthase; C2MC: Chromosome 2 miRNA cluster; DMEM: Dulbecco’s modified Eagle medium; eWAT: epididymal white adipose tissue; ETC: electron transport chain; FAO: fatty acid oxidation; *Fabp4*:fatty acid binding protein 4; FBS: fetal bovine serum; *Hadha*: hydroxyacyl-CoA dehydrogenase trifunctional multienzyme complex subunit alpha; *Hadhb*: hydroxyacyl-CoA dehydrogenase trifunctional multienzyme complex subunit beta; HFD: high fat diet; *Idh3a*: isocitrate dehydrogenase 3 alpha; iWAT: inguinal subcutaneous white adipose tissue; *Lpl*: lipoprotein lipase; *Mdh2*: malate dehydrogenase 2; NBCS: NewBorn Calf Serum; *mt-Nd1*: mitochondrial NADH dehydrogenase 1; *Ndufb8*:ubiquinone oxidoreductase subunit B8; *Nrf1*: nuclear respiratory factor 1; *Pgc1α*: peroxisome proliferative activated receptor gamma coactivator 1 alpha; *Pgc1b*: peroxisome proliferative activated receptor, gamma, coactivator 1 beta; *Pparγ*: peroxisome proliferator activated receptor gamma; *Prdm16*: PR domain containing 16; *Rgs4*: regulator of G-protein signaling 4; *Sdhb*: succinate dehydrogenase complex, subunit B; *Sdhc*: succinate dehydrogenase complex, subunit C; *Sdhd*: succinate dehydrogenase complex, subunit D; *Sh3d21*: SH3 domain containing 21; *Sfmbt2*: Scm-like with four mbt domains 2; TG: triglyceride; TCA: tricarboxylic acid cycle; *Tfam*: transcription factor A, mitochondrial; TMRE: tetramethylrhodamine, methyl ester; *Ucp1*: uncoupling protein 1; *Uqcrc2*: ubiquinol cytochrome c reductase core protein 2; WAT: White adipose tissue

## Introduction

Obesity is the single biggest risk factor in a multitude of metabolic diseases, including certain cancers, diabetes, and cardiovascular disease [1,2]. The dramatic rise in obesity has put a serious strain on public health networks across the globe [3]. Despite nutritional interventions and physical education programmes, the prevalence of obesity is still increasing and ~600 million people worldwide are expected to be obese by 2025, according to World Health Organization (WHO) estimations [4]. Therefore, a thorough understanding of the mechanisms that regulate adipogenesis could have clinical relevance in preventing and treating obesity and the associated metabolic syndromes.

White adipose tissue (WAT) and brown adipose tissue (BAT) are the two principal types of adipose tissues in mammals. WAT primarily serves to store extra energy as triglycerides, while BAT is specialized to burn lipids for heat generation and energy expenditure as a defence against cold and obesity [5]. The development of WAT browning and BAT activation is dramatically enhanced during adaptation to cold or in response to treatment with β3-selective adrenergic agonists [6,7]. Accordingly, promoting BAT-like features in white adipose and BAT function is an attractive therapeutic approach to combat obesity [8].

MicroRNAs (miRNAs) are a family of 21–25 nucleotide small RNAs that negatively regulate gene expression at the post-transcriptional level. miRNAs exhibit temporally and spatially regulated expression patterns during diverse developmental and physiological processes [9–11]. Several miRNAs have recently emerged as important regulators of either brown adipocyte differentiation or white adipocyte browning. The *miR-193b-365* cluster was the first reported miRNAs that sustain brown adipocyte differentiation by repressing the myogenic potential of preadipocytes [12]. Overexpression of the miR17-92 cluster in C3H10T1/2 cells enhances the thermogenic capacity of adipocytes, and there is a significant reduction in adiposity in adipose tissue-specific miR17-92 cluster transgenic mice as well [13]. Several miRNAs also negatively regulate brown fat development. *miR-133* directly targets and negatively regulates *Prdm16*, and inhibition of *miR-133* promotes differentiation of precursors from BAT and WAT to mature brown adipocytes [14]. A study reported that decreased *miR-494-3p* expression during browning regulates mitochondrial biogenesis and thermogenesis through PGC1-α in beige adipocytes [15]. Despite the fact that some miRNAs have been identified as central regulators of the brown adipogenic programme, the whole miRNA regulatory network is still not complete, and further studies are required to fully understand the regulatory roles of miRNAs in brown adipogenesis and to develop therapeutic approaches to treat obesity and obesity-related diseases.

**Figure 1.**
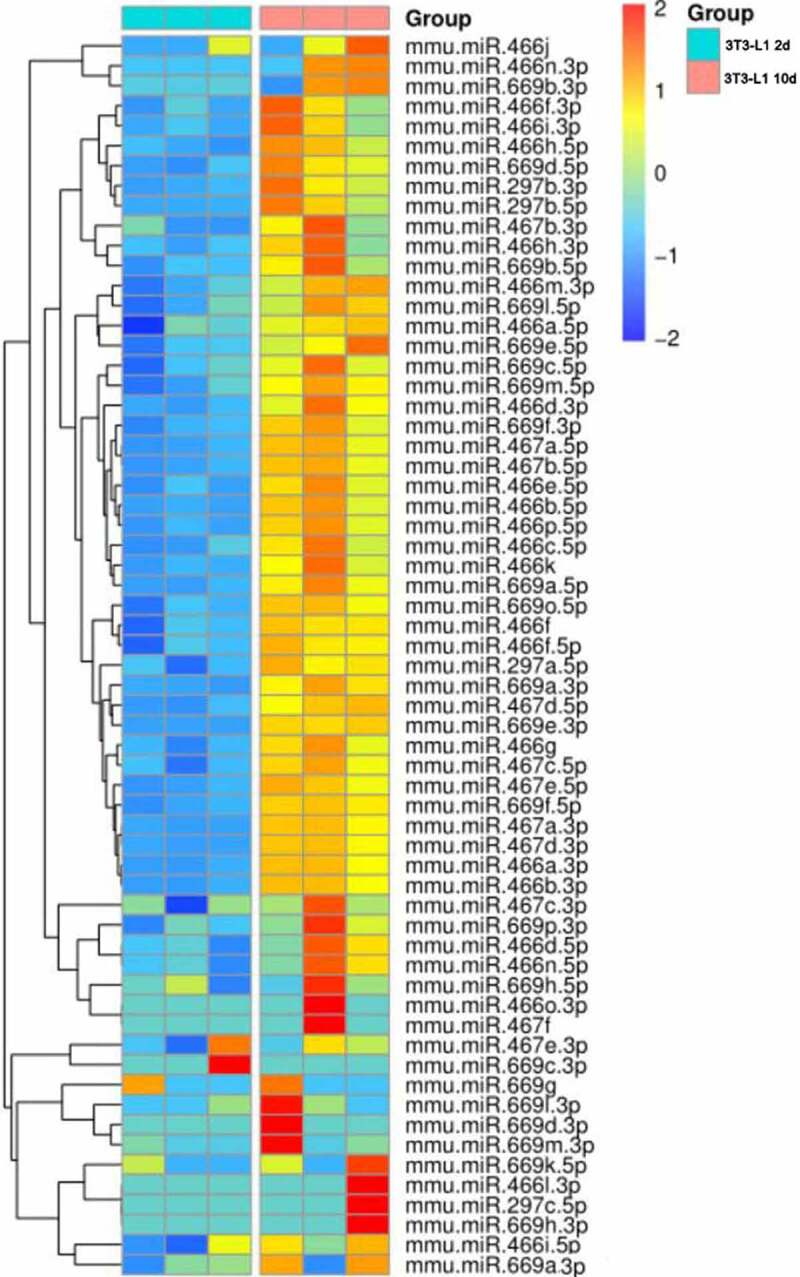
Heatmap of C2MC expression in the mature 3T3-L1 adipocytes (day 10) and 3T3-L1 preadipocyte (day 2).

Preadipocytes are often used as models for elucidating the underlying mechanisms of adipogenesis, which involves cell proliferation and differentiation. To identify the miRNAs related to lipid metabolism, we analysed RNA isolated from 3T3-L1 preadipocytes at different time points of differentiation, from preadipocytes to mature. microRNA sequencing data showed that most members of the *miR 297–466-467-699* cluster are upregulated during 3T3-L1 adipocyte differentiation, indicating that the *miR 297–466-467-699* cluster likely regulates adipocyte differentiation and lipid metabolism (Figure 1). In addition, a previous study has shown that *miR-669a-5p* has a close relationship with progenitor cell differentiation [16], so we focused on *miR-669a-5p* to explore its role in preadipocytes.

*miR-669a-5p* is a member of the *miR 297–466-467-699* cluster located in intron 10 of the *Sfmbt2* gene on mouse chromosome 2 (hence *Sfmbt2* miRNA cluster is also called Chromosome 2 miRNA cluster, C2MC). C2MC is one of the largest clusters of miRNAs, containing 72 miRNA precursor sequences [17], and these C2MC sequences show a high degree of similarity [18]. However, little is known about the biological functions of the C2MC biological functions. A small number of manuscripts have shown that C2MC miRNA members play important roles in cell differentiation and apoptosis [16,19–22], as well as in development and the immune response [17,23–25].

In this report, we determine the role of *miR-669a-5p* in adipogenesis using a mouse 3T3-L1 embryonic fibroblastic cell line and a C3H10T1/2 mesenchymal stem cell line. We describe the expression profiles of *miR-669a-5p* during adipogenic differentiation and demonstrate that *miR-669a-5p* enhances adipogenic differentiation and induces a brown adipocyte-like phenotype in 3T3-L1 and C3H10T1/2 preadipocytes. It is the first study that demonstrates the function of *miR-669a-5p* in adiposeness. It is also the first study to show that *Sfmbt2* and C2MC are cotranscripted during adipogenic differentiation in 3T3-L1 and C3H10T1/2 cells, indicating that *Sfmbt2* and C2MC may act in concert to regulate adipogenic differentiation.

## Results

### The expression of miR-669a-5p is increased during differentiation of 3T3-L1 cells

To identify miRNAs involved in the adipogenesis process, we performed small RNA deep sequencing in 3T3-L1 cells undergoing differentiation. miRNA-Seq analysis indicates C2MC may regulate lipid metabolism: Nearly 90% of the members of the *miR 297–466-467-699* cluster are upregulated in mature 3T3-L1 adipocytes (day 10) compared to 3T3-L1 preadipocytes (day 2) (Figure 1). *miR-669a-5p i*s a member of C2MC, and a previous study has shown *miR-669a-5p* plays a role in the process of progenitor cells differentiation [16], but whether it impacts preadipocytes differentiation and adipogenesis has not been characterized.

**Figure 2.**
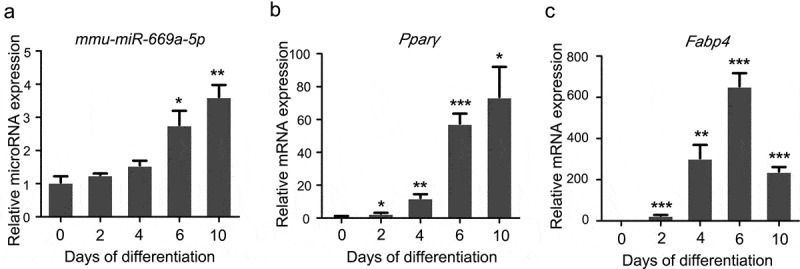
*miR-669a-5p* is increased during differentiation of 3T3-L1 cells. (a)RT-qPCR to analyse the expression of *miR-669a-5p* in 3T3-L1 cells during differentiation, normalized to *U6* expression. n = 3 per group.(b and c) RT-qPCR to analyse the mRNA expression levels of adipogenesis markers *Pparγ* and *Fabp4* in 3T3-L1 cells during differentiation, normalized to *β-actin* expression. n = 3 per group.Data are representative of at least three individual experiments. Results are represented as mean ± SEM. *p < 0.05, **p < 0.01, ***p < 0.001 versus day 0 group.

To determine the role of *miR-669a-5p* in preadipocytes differentiation and adiposenesis, we first detected the levels of *miR-669a-5p* during 3T3-L1 white preadipocytes differentiation. Cells were collected at the indicated time points of differentiation (day 0, day 2, day 4, day 6, day 8 and day 10 after adipogenic treatment). *miR-669a-5p* was significantly increased during the adipogenic differentiation process (Figure 2(a)). Differentiation of the cells was verified by measuring the mRNA expression of the adipogenic markers peroxisome proliferator activated receptor gamma (*Pparγ*) and fatty acid binding protein 4 (*Fabp4*) (Figure 2(b) and (c)). Taken together, these data indicate that *miR-669a-5p* may play a role in adipocyte differentiation.

### miR-669a-5p promotes the differentiation of 3T3-L1 cells

**Figure 3.**
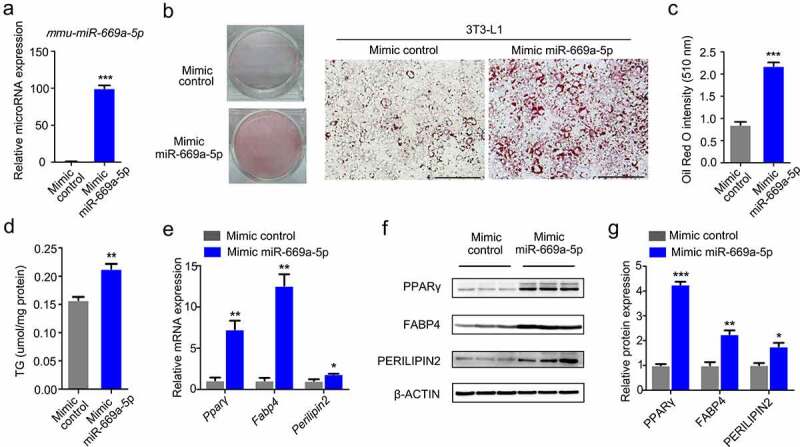
*miR-669a-5p* promotes the differentiation of 3T3-L1 cells. 3T3-L1 preadipocytes were transfected with mimic control or mimic miR-669a-5p (100 nM) on day 0 and day 4 after differentiation, cells were harvested on day 8 for analysis.(a) RT-qPCR to analyse the expression of *miR-669a-5p* in 3T3-L1 cells transfected with mimic control or mimic *miR-669a-5p* during differentiation, normalized to *U6* expression. n = 3 per group.(b-c) Lipid accumulation was assessed by Oil Red O staining, and the absorbance was measured at 510 nm wave length. A representative image of three independent experiments is shown in B.(d) Lipid accumulation was assessed by intracellular TG content. n = 3 per group(e) RT-qPCR to analyse the mRNA levels of adipogenesis markers *Pparγ, Fabp4* and *perilipin2* in differentiated 3T3-L1 adipocytes transfected with mimic control or mimic *miR-669a-5p*, normalized to *β-actin* expression. n = 3 per group.(f) Western blot to evaluate the protein levers of adipogenesis markers PPARγ, FABP4 and PERILIPLIN2 in differentiated 3T3-L1 adipocytes transfected with mimic control or mimic *miR-669a-5p*, β-ACTIN was used as a loading control. n = 3 per group.(g) Quantitative densitometry of the Western blots showed in F.Data are representative of at least three individual experiments. Results are represented as mean ± SEM. **p < 0.01 versus mimic control group. Scale bar indicates 200 μm in B.

To further elucidate the role of *miR-669a-5p* during adipogenesis, we transiently transfected 3T3-L1 cells with a *miR-669a-5p* mimic or inhibitor at different time points during 3T3-L1 differentiation. The level of *miR-669a-5p* was increased when the *miR-669a-5p* mimic was transfected into 3T3‐L1 preadipocytes (Figure 3(a)). Oil Red O staining and quantification of intracellular triglyceride demonstrated that supplement with the *miR-669a-5p* mimic increased lipid droplet formation in 3T3-L1 (Figure 3(b-d)). Consistently, the expression levels of the adipogenic marker genes *Pparγ, Fabp4* and *Perilipin2* were significantly upregulated after a *miR-669a-5p* mimic treatment (Figure 3(e-g)). These results suggest that *miR-669a-5p* promotes 3T3-L1 preadipocytes differentiation.

The effect on adipogenesis of a *miR-669a-5p* inhibitor was also examined in the 3T3-L1 cell. Transfection of the *miR-669a-5p* inhibitor decreased *miR-669a-5p* expression (Figure S1A). However, lipid droplet formation and adipogenic markers expression did not show a significant change following the *miR-669a-5p* inhibitor transfection (Figure S1B-F).

### miR-669a-5p promotes adipogenic differentiation of C3H10T1/2 cells

**Figure 4.**
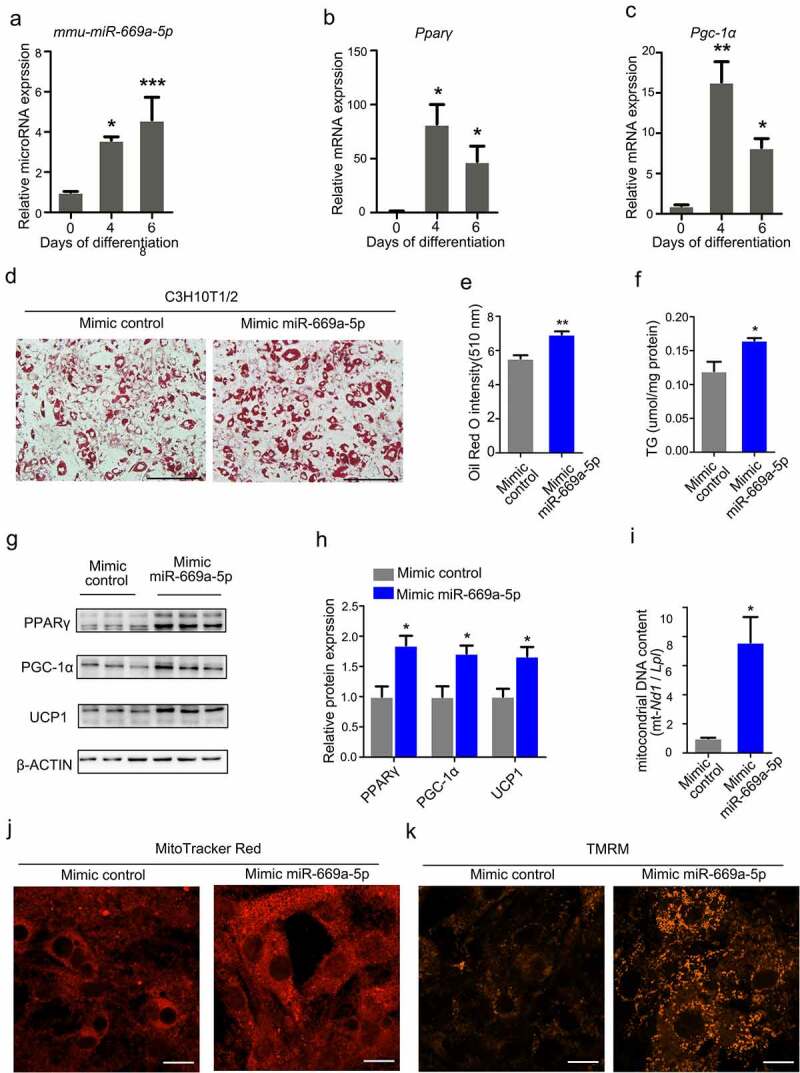
*miR-669a-5p* promotes adipogenic differentiation of C3H10T1/2 cells. C3H10T1/2 preadipocytes were transfected with mimic control or mimic miR-669a-5p (100 nM) on day 0 and day 3 after adipogenic differentiation, the cells were collected on day 6 for analysis.(a-c) RT-qPCR to analyse the expression of *miR-669a-5p* and brown fat-related genes (*Pparγ* and *Pgc1α*) during C3H10T1/2 differentiation, normalized to *U6* or *β-actin* expression. n = 3 per group.(d-e) Lipid accumulation was assessed by Oil-Red O staining, and the absorbance was measuredat 510 nm wave length. A representative image of three independent experiments is shown in D.(f) Lipid accumulation was assessed by intracellular TG content. n = 3 per group(g) Western blot to evaluate the protein expression of brown fat genes PPARγ, PGC-1α and UCP1, β-ACTIN was used as a loading control. n = 3 per group.(h) Quantitative densitometry of the Western blots showed in F.(i) The quantification of mitochondrial DNA content. n = 3 per group(j) Mitochondrial mass was measured by MitoTracker Red staining on day 6. n = 3 per group.(k) Mitochondrial membrane potential was measured by TMRM on day 6. n = 3 per group.Data are representative of at least three individual experiments. Results are represented as mean ± SEM. *p < 0.05, **p < 0.01, ***p < 0.001 versus mimic control group. Scale bar indicates 200 μm in D. Scale bar indicates 20 μm in J and K.

To further demonstrate the role of *miR-669a-5p* in adipogenesis, we tested *miR-669a-5p* expression levels at different time-points during adipogenesis of the brown preadipocyte cell line C3H10T1/2. During the differentiation process of this cell line, a thermogenic programme is induced and the levels of brown fat-related genes (also called thermogenic genes) such as uncoupling protein 1(*Ucp1*) and peroxisome proliferative activated receptor gamma coactivator 1 alpha *(Pgc-1a*) are increased. The levels of *miR-669a-5p* and the brown fat-related genes *Pparγ* and *Pgc-1α* were increased in C3H10T1/2 cells after the adipogenic differentiation treatment (Figure 4(a-c)). In addition, the adipogenic differentiation was accelerated by supplementing a *miR-669a-5p* mimic, as evidenced by the Oil Red O staining, measurement of the intracellular triglyceride (TG) content and Western blot analysis of brown fat-related genes (PPARγ, PGC-1α and UCP1)(Figure 4(d-h)). Consistent with the gene expression data, *miR-669a-5p* increased the mitochondrial content as shown by mtDNA quantification and Mito Tracker staining (Figure 4(i) and (j)). The mitochondrial membrane potential was also measured by TMRM, the results showed *miR-669a-5p* increased the mitochondrial membrane potential of C3H10T1/2 cells (Figure 4(k)).

Also consistent with 3T3-L1 data, the *miR-669a-5p* inhibitor had no effect on lipid droplet formation and brown fat-related genes expression during adipogenic differentiation of C3H10T1/2 cells (Figure S2). These results show that *miR-669a-5p* stimulates adipogenic differentiation in 3T3-L1 cells and C3H10T1/2 cells, and the effects of the *miR-669a-5p* inhibitor were not observed in either cell line.

### miR-669a-5p induces a brown phenotype in 3T3-L1 adipocytes

**Figure 5.**
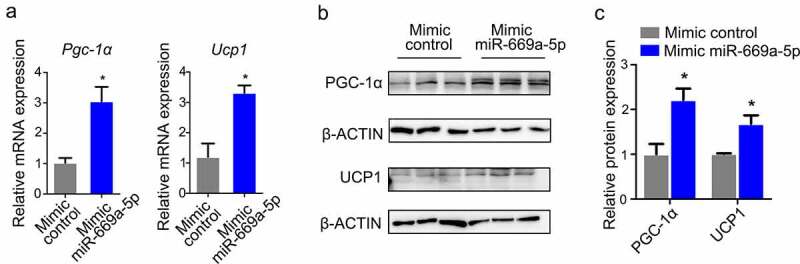
*miR-669a-5p* induces a brown adipocyte phenotype in 3T3-L1 adipocytes. 3T3-L1 preadipocytes were transfected with mimic control or mimic miR-669a-5p (100 nm) on day 0 and day 4 after differentiation, cells were harvested on day 8 for analysis.(a) RT-qPCR to analyse the expression of thermogenic genes *Pgc-1α* and *Ucp1* in 3T3-L1 adipocytes, normalized to *β-actin* expression. n = 3 per group.(b) Western blot to evaluate the protein levels of thermogenic genes PGC-1α and UCP1 in 3T3-L1 adipocytes, β-ACTIN was used as a loading control. n = 3 per group.(c) Quantitative densitometry of the Western blots showed in B.Data are representative of at least three individual experiments. Results are represented as mean ± SEM. *p < 0.05 versus mimic control group.

Given the prominent effect of *miR-669a-5p* on the development of the thermogenic programme in brown adipocytes C3H10T1/2 (Figure 4), we asked whether *miR-669a-5p* also has similar effects in the white adipocyte 3T3-L1 cells. We tested the expression of thermogenic genes in differentiated 3T3-L1 transfected with the *miR-669a-5p* mimic. As shown in Figure 5, the *miR-669a-5p* mimic significantly enhanced the expression of the thermogenic marker genes *Pgc-1α* and *Ucp1* in 3T3-L1 cells (Figure 5(a-c)), suggesting that *miR-669a-5p* induces a brown phenotype in 3T3-L1 adipocytes.

### miR-669a-5p promotes mitochondrial biogenesis 3T3-L1 cells

To illustrate the mechanisms by which *miR-669a-5p* controls adipogenesis, we conducted a transcriptome study by RNA-seq and quantified the mRNA changes during differentiation of 3T3-L1 cells transfected with the *miR-669a-5p* mimic transfection. Increased mitochondrial biogenesis is one of the characteristics in the adipocyte browning process. Our mRNA-seq data show that the transcripts regulating mitochondriogenesis are enhanced in the presence of the *miR-669a-5p* mimic (Figure S3).

**Figure 6.**
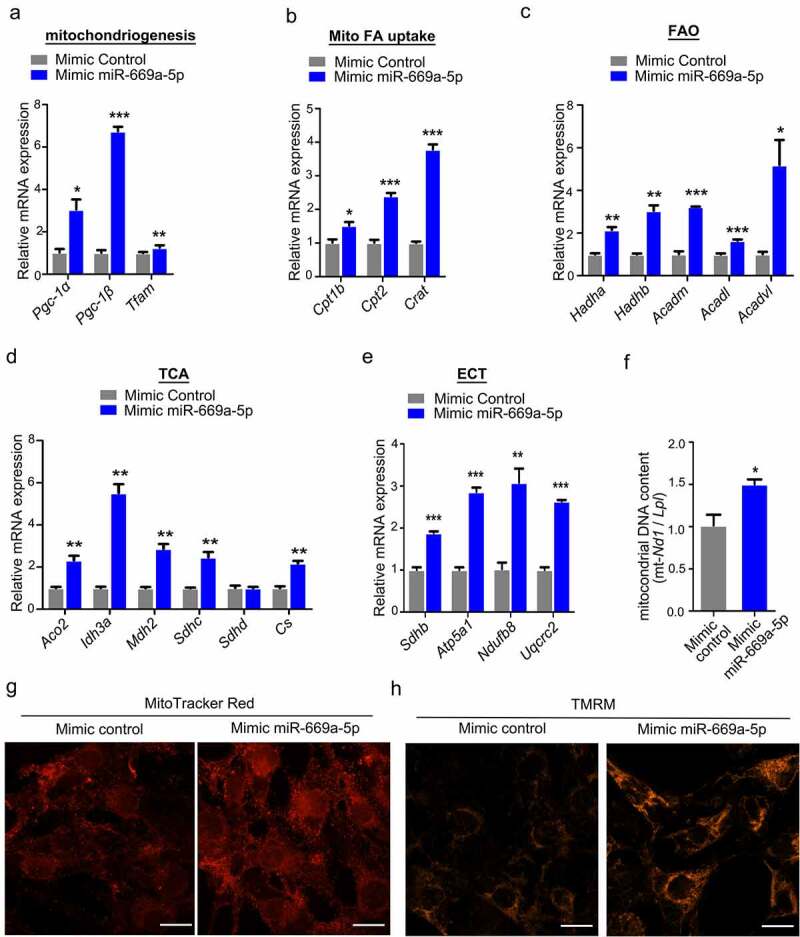
*miR-669a-5p* enhances the mitochondrial biogenesis in 3T3-L1 cells. 3T3-L1 preadipocytes were transfected with mimic control or mimic miR-669a-5p (100 nM) on day 0 and day 4 after differentiation, the cells were collected on day 8 for analysis.(a) RT-qPCR analysis of mitochondrial genes involved in mitochondriogenesis, normalized to *β-actin* expression. n = 3 per group.(b) RT-qPCR analysis of mitochondrial genes involved in fatty acid uptake (Mito FA uptake) in mature adipocytes, normalized to *β-actin* expression. n = 3 per group.(c) RT-qPCR analysis of mitochondrial genes involved in β-oxidation (FAO) in mature adipocytes, normalized to *β-actin* expression. n = 3 per group.(d) RT-qPCR analysis of representative tricarboxylic acid (TCA) cycle genes in mature adipocytes, normalized to *β-actin* expression. n = 3 per group.(e) RT-qPCR analysis of representative genes of the five complexes of the mitochondrial electron transport chain (ETC) in mature adipocytes, normalized to *β-actin* expression. n = 3 per group.(f) The quantification of mitochondrial DNA content. n = 3 per group(g) Mitochondrial mass was measured by MitoTracker Red staining on day 8. n = 3 per group.(h) Mitochondrial membrane potential was measured by TMRM on day 8. n = 3 per group.Data are representative of at least three individual experiments. Results are represented as mean ± SEM. *p < 0.05, **p < 0.01, ***p < 0.001 versus mimic control group. Scale bar indicates 20 μm in G and H.

We next performed RT-qPCR to validate the transcript level of those key genes related to mitochondria. We found that *miR-669a-5p* treatment increased the expression of genes (*Pgc-1a, Pgc-1β, Tfam*) known to be involved in mitochondriogenesis (Figure 6(a)). A *miR-669a-5p* mimic treatment also led to an increased expression of the mitochondrial free fatty acid transporter machinery (*Cpt1b, Cpt2*, and *Crat*) (Figure 6(b)). In addition, the expression of all components of the *β*-oxidation machinery and most genes of the tricarboxylic acid cycle (TCA) were upregulated by the *miR-669a-5p* mimic treatment (Figure 6(c) and (d)). Since the electron transport chain (ETC) is the final acceptor of the co-factors produced by fatty acid oxidation (FAO) and TCA, we quantified the expression of different components of the ETC. Transfection of the *miR-669a-5p* mimic led to an increase in mRNA expression of members in all five complexes of the ETC (Figure 6(e)).

We further tested the mitochondrial content in differentiated 3T3-L1 cells transfected with the *miR-669a-5p* mimic. We found that the *miR-669a-5p* mimic markedly increased mitochondrial content indicated by mtDNA quantification and Mito Tracker staining (figure 6(f) and (g)). To illustrate the mitochondrial activity, the mitochondrial membrane potential was measured by TMRM. The results showed *miR-669a-5p* increased the mitochondrial membrane potential of 3T3-L1 cells (Figure 6(h)).

Taken together, our results reveal that *miR-669a-5p* positively regulates the bioenergetics of 3T3-L1 cells by acquisition of a mature brown fat phenotype.

### The expression of miR-669a-5p is upregulated in iWAT of mice exposed to cold

Since *miR-669a-5p* promotes adipogenesis in preadipocytes, we used diet-induced obesity (DIO) mice as an obesity model to investigate the function of *miR-669a-5p in vivo*. After being fed with a high-fat diet for 2 months (from week 8 to week 16), the body weight and body fat ratio of DIO mice were significantly increased compared with littermate controls (Figure S4A). There were no significant expression changes of *miR-669a-5p* in both Epididymal white adipose tissue (eWAT) and BAT of DIO mice. However, we found a declining trend of *miR-669a-5p* expression in eWAT of DIO mice (Figure S4B), which indicates *miR-669a-5p* may participate in the process of WAT browning.

**Figure 7.**
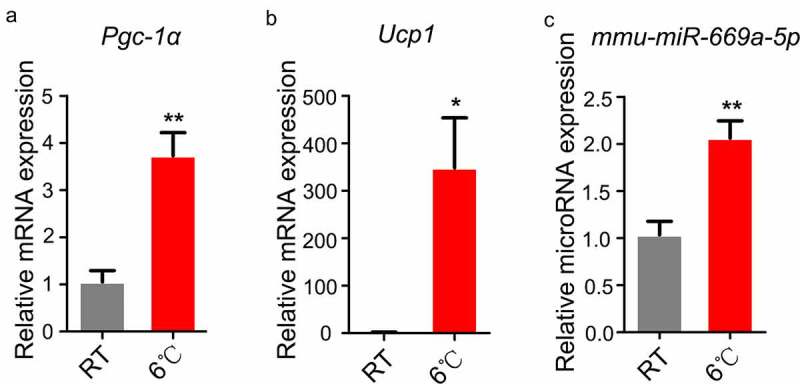
The expression of *miR-669a-5p* is upregulated in iWAT of mice exposed to cold. (a-b) RT-qPCR to analyse the expression of thermogenic genes *Pgc-1α* and *Ucp1* in iWAT from mice at room temperature (RT) or post cold exposure, normalized to *β-actin* expression. n = 5 per group.(c) RT-qPCR to analyse the expression of *miR-669a-5p* in iWAT from mice at room temperature (RT) or post cold exposure, normalized to *U6* expression. n = 5 per group.Data are representative of at least three individual experiments. Results are represented as mean ± SEM. *p < 0.05, **p < 0.01 versus RT group.

Given the impact of *miR-669a-5p* on the development of a brown fat-like thermogenic programme in white adipocytes (Figures 5,6), we studied whether the expression of *miR-669a-5p* is altered in a cold-induced WAT browning mice model which is a more widely used animal model for the research of WAT browning. Inguinal subcutaneous white adipose tissue (iWAT) is a typical adipocyte tissue that undergoes WAT browning process when mice are exposed to cold. After exposure to cold, we found a stronger induction of *Ucp1* and *Pgc-1α* expression in iWAT (Figure 7(a,b)). More importantly, we found *miR-669a-5p* was increased in the iWAT of mice subjected to cold exposure, suggesting that *miR-669a-5p* participates in the process of WAT browning *in vivo* (Figure 7(c)).

## Discussion

Obesity is characterized by increased lipid storage in adipocytes and an increased number of adipocytes. Adipocytes are derived from a pool of existing preadipocytes, which are ready to differentiate in response to an appropriate signal. This process has produced intense research into the mechanisms regulating adipocyte development [26]. Mesenchymal stem cell-line C3H10T1/2 and mouse 3T3-L1 preadipocytes are often used as models for elucidating the underlying mechanisms of adipogenesis, which involves cell proliferation and differentiation [27–30].

Recent data indicate that microRNAs play a key role in modulating cell differentiation and metabolism of many tissue types *in vivo*, including adipocytes [12]. MicroRNAs that selectively promote energy expenditure through BAT thermogenesis or browning of WAT are of significant interest as potential therapeutics for anti-obesity treatments due to the ability of BAT and browning WAT to metabolize vast amounts of glucose and lipids [7,8].

In this study, we demonstrate the function of *miR-669a-5p* in adipogenesis. We show that the expression of *miR-669a-5p* is increased during adipogenic differentiation in 3T3-L1 and C3H10T1/2 cells. Meanwhile, we further show that *miR-669a-5p* is able to stimulate adipogenesis and induces a brown phenotype in 3T3-L1 and C3H10T1/2 cells. In line with the *in vitro* data, the expression of *miR-669a-5p* is upregulated in iWAT when mice are exposed to cold. Our data indicate that *miR-669a-5p* participates in the process of adipose tissue browning both in *vivo* and *vitro*. However, a *miR-669a-5p* inhibitor has no effect on adipogenic differentiation in 3T3-L1 and C3H10T1/2 cells. This result indicates that the function of *miR-669a-5p* in adipogenesis is not indispensable. As the sequences of C2MC show high degrees of similarity, the function of *miR-669a-5p* may be compensated by other C2MC members which have similar sequences with *miR-669a-5p*.

According to our previous studies and mRNA-seq data, we also tried to find the target of *miR-669a-5p* in the process of adipocyte differentiation. We have tested some genes, such as *Fst, Vdr, Ctbp2*, ect. (data not shown). However, we have not found the target of *miR-669a-5p*. It will be interesting to find the underlying mechanisms in the future.

Previous studies have shown that clustered miRNAs often coexpressed with their host genes [31–33], with this the expression pattern serving to regulate similar biological functions and signalling pathways. We found that the expression of *Sfmbt2* and C2MC were increased during adipogenic differentiation in 3T3-L1 and C3H10T1/2, indicating *Sfmbt2* and C2MC are cotranscripted (Figure S5 and S6, Figures 2(a) and 4(a)). The coexpression patterns and high sequence similarity of miRNA family members suggest that *Sfmbt2* and C2MC may act in concert to regulate adipogenic differentiation in 3T3-L1 and C3H10T1/2 cells. In the present study, we focus on the function of *miR-669a-5p*. Further studies are needed to verify the function of the other *miR 297–466-467-699* cluster members and to elucidate the role of the *Sfmbt2* gene in the process of adipogenesis. As well, our findings about *miR-669a-5p* need more mechanistic investigations and verifications through *in vitro and vivo* experiments.

In summary, we have demonstrated for the first time that the expression of *miR-669a-5p* is increased during adipogenic differentiation of 3T3-L1 preadipocytes and C3H10T1/2 cells. In addition, *miR-669a-5p* is able to stimulate adipogenesis and induce a brown phenotype in 3T3-L1 and C3H10T1/2 cells.

## Materials and methods

### RNA sequencing

Small RNA sequencing libraries were generated using NEBNext®Multiplex Small RNA Library Prep Set for Illumina® (NEB) and the clustering of the index-coded samples was performed on a cBot Cluster Generation System using TruSeq SR Cluster Kit v3-cBot-HS (Illumia) according to the manufacturer’s instructions. After cluster generation, the library preparations were sequenced on an Illumina Hiseq 2500/2000 platform and 50 bp single-end reads were generated.

mRNA sequencing libraries were generated using NEBNext® UltraTM RNA Library Prep Kit for Illumina® (NEB) following manufacturer’s recommendations. The library preparations were sequenced on an Illumina Novaseq platform and 150 bp paired-end reads were generated. Gene Ontology (GO) enrichment analysis of differentially expressed genes was implemented by the clusterProfiler R package, in which gene length bias was corrected. GO terms with corrected *p* value less than 0.05 were considered significantly enriched by differential expressed genes.

### Animal studies

Male C57BL/6 mice were kept under constant temperature and humidity in a 12 h controlled dark/light cycle. 8-week-old male C57BL6/J mice were fed with a standard chow diet (10 kcal% fat, D12450J, Research Diets) or high fat diet (HFD, 60 kcal% fat, D12492, Research Diets) for 8 weeks. Mice were then euthanized and different adipose tissues were harvested for analysis. For cold-exposure experiments, 8-week-old male mice were exposed to 6°C for 72 h. All of the experiments were approved by the by the Animal Ethics Committee of the Kunming Institute of Zoology, Chinese Academy of Science.

### 3T3-L1 induction differentiation

The 3T3-L1 mouse preadipocyte line was cultured in Dulbecco’s modified Eagle medium (DMEM; C11995500CP, Gibco) with 10% Newborn calf serum (NBCS; 16,010–159, Gibco) at 37°C and 5% CO2 in a humidified atmosphere. Differentiation was initiated 2 days post-confluence (day 0), cells were induced to differentiate for 3 days with DMEM containing 10% foetal bovine serum (FBS; 10,099–141 C, Gibco), 170 nM insulin (11,376,497,001, Roche), 1 uM dexamethasone (60231A, Adamas), 500 uM isobutylmethylxanthine (IBMX; I5879, Sigma), and 1 uM Rosiglitazone (R2408, Sigma). After these 3 days, the medium was replaced with DMEM containing 10% FBS and 170 nM insulin for 1 day, then cells were incubated in DMEM with 10% FBS for an additional 5 days. 3T3-L1 preadipocytes that differentiated to adipocytes were stained with Oil-Red and detected by light microscopy.

### C3H10T1/2 induction differentiation

The C3H10T1/2 cell line was cultured in DMEM containing 10% FBS at 37°C and 5% CO2 in a humidified atmosphere. Cells were cultured to confluence then exposed to differentiation media (day 0): DMEM, 10% FBS, 20 nM insulin, 1 nM triiodothyronine (T3; T2877-100, Sigma), 500 uM IBMX, 1 uM dexamethasone and 125 uM indomethacin (I7378, Sigma) for 2 days. Cells were then switched to media containing DMEM, 10% FBS, 20 nM insulin and 1 nM T3 for 4 days until the adipocytes matured. C3H10T1/2 mesenchymal stem cells that differentiated to adipocytes were stained with Oil-Red and detected by light microscopy.

### Cell transfection

Mimic and inhibitor oligonucleotides of *miR-669a-5p* were synthesized by Ribobio. The transfection was carried out using the riboFECT^TM^ Transfection Kit (C10511-05, Ribobio). According to the manufacturer’s instructions, cells grown in standard 6-well plates were added with a mixture of mimic (200 nM) or inhibitor (100 nM). Transfection was carried out two times during the cell differentiation.3T3-L1 preadipocytes were transfected with mimic control/inhibitor control or mimic *miR-669a-5p* (100 nM)/inhibitor *miR-669a-5p* (200 nM) on day 0 and day 4 after differentiation, cells were harvested on day 8 for analysis. C3H10T1/2 preadipocytes were transfected with mimic control/inhibitor control or mimic *miR-669a-5p* (100 nM)/ inhibitor *miR-669a-5p* (200 nM) on day 0 and day 3 after adipogenic differentiation. The cells were collected on day 6 for analysis

### Oil Red O staining and measurement of intracellular triglyceride (TG) content

Accumulation of lipids was assessed by oil red O staining. Cells were washed with PBS and fixed with 4% paraformaldehyde for 1 h at room temperature. 60% isopropanol was then added to the cells for 2 min. Cells were then incubated with freshly prepared oil red O working solution for 30

min at 37°C and observed under the microscope. For Oil Red O quantitative analysis, the intracellular absorbed Oil Red O was extracted with 100% isopropanol, and absorbance was measured at 510 nm wave length. Intracellular TG contents were measured using a commercial assay kit (E1013, APPLYGEN), TG concentrations were calculated based upon a standard curve made from TG standards and normalized to total cellular protein content.

### Reverse transcription quantitative real-time polymerase chain reaction (RT- qPCR)

Total RNA was isolated from the samples using RNAiso Plus (9109, Takara). The expression levels of miRNA were analysed using an All-in-One™ miRNA qRT-PCR Detection Kit (QP015, GeneCopoeia). For mRNA expression analysis, total RNA was transcribed to complementary DNA using PrimeScript RT reagent Kit with gDNA Eraser (RR047A, Takara) following the manufacturer’s instructions. Expression of selected genes was analysed using *TransStart*® Top Green qPCR SuperMix (AQ132, TransGen Biotech). Quantitative real-time polymerase chain reaction (qPCR) was performed on the ABI-7900HT system (Applied Biosystems). The relative expression of target genes was measured using the 2^−ΔΔCt^ method, and the amount of target gene was normalized to the endogenous control gene *U6* or *β-actin*. Sequences of primers are shown in Table S1.

### Total DNA isolation and mtDNA quantification

Total DNA was isolated from cells using the Blood/Cell/tissue genomic DNA extraction kit (DP304, TIANGEN). The mtDNA copy number quantification was performed by real-time PCR. Amplification of the mitochondrial mt-*Nd1* gene was compared with that of reference nuclear *Lpl*. Primers used are shown in Table S1.

### Western blotting

Samples were lysed in RIPA buffer (P0013B, Beyotime) containing protease inhibitor cocktail (P8340, Sigma) and PMSF (A610425-0005, Sangon Biotech). Proteins were separated by SDS–PAGE and transferred onto polyvinylidene difluoride membranes. Blocking and antibody incubations were performed in 5% BSA or non-fat dry milk. Anti- PPARγ (16,643-1-AP, 1:1000), anti-FABP4 (12,802-1-AP, 1:1000), anti-PERILIPIN2 (15,294-1-AP), anti-PGC1α (66,369-1-Ig) and anti-UCP1 (23,673-1-AP) were purchased from Proteintech. Anti-β-actin (A5441, 1:1500) was purchased from Sigma-Aldrich. Antibody detection reactions were developed by enhanced chemiluminescence reagent (Millipore) and imaged using the MiniChemi 610 imaging system (Sage Creation). Quantification was done using Lane 1D software (Sage Creation).

### Mitochondrial labelling

Mitochondrial mass was measured by fluorescence levels on staining with MitoTracker Red CMXRos (M7512, Invitrogen). Cells were incubated with 50 nM MitoTracker Red CMXRos for 10 min at 37°C. Cells were then fixed with 3.7% formaldehyde. The images were captured with a ZEISS LSM800 confocal microscope.

### Mitochondrial membrane potential measurement

To measure the mitochondrial membrane potential, adipocytes were stained with cell growth medium containing 100 nM tetramethylrhodamine, methyl ester (TMRM, I34361, Invitrogen). Cells were then incubated for 30 min at 37°C. The images were captured with a ZEISS LSM800 confocal microscope.

### Statistical analysis

All results are presented as mean ± standard errors of the means (SEM) based on at least three separate experiments. Differences between two groups were analysed by Student’s *t*-test using GraphPad Prism 5.0. *p* < 0.05 was considered statistically significant.

## Supplementary Material

Supplemental Material

## Data Availability

The data that support the findings of this study are available from the corresponding author upon reasonable request
