# Supplementary material for: miR-669a-5p promotes adipogenic differentiation and induces browning in preadipocytes
Source: Adipocyte. 2022 Jan 30;11(1):120–32. doi: 10.1080/21623945.2022.2030570 (PMC8803067; doi:10.1080/21623945.2022.2030570)
Supplement: Supplemental Material [file KADI_A_2030570_SM9851.zip › supplementary/supplymental material Figure ligends.docx]

**Supplementary data to:**

***miR-669a-5p* promotes adipogenic differentiation and induces browning in preadipocytes**

**Figure Legends**

**Figure S1. *miR-669a-5p* inhibitor does not suppress the differentiation of 3T3-L1 cells differentiation**

3T3-L1 preadipocytes were transfected with inhibitor control or inhibitor *miR-669a-5p* (200 nM) on day 0 and day 4 after differentiation, the cells were collected on day 8 for analysis.

(A) RT-qPCR to analyze the expression of *miR-669a-5p* in 3T3-L1 cells transfected with inhibitor control or inhibitor *miR-669a-5p* during differentiation, normalized to *U6* expression. n = 3 per group.

(B-C) Lipid accumulation was assessed by Oil Red O staining, and the absorbance was measured at 510 nm wave length. The representative image of three independent experiments is shown in B.

(D) RT-qPCR was performed to measure the mRNA levels of adipogenesis markers *Pparγ* and *Fabp4*, normalized to *β-actin* expression. n = 3 per group.

(E) Western blot was performed to determine the protein levels of adipogenesis markers PPARγ and FABP4, β-ACTIN was used as a loading control. n = 3 per group.

(F) Quantitative densitometry of the western blots showed in E.

Data are representative of at least three individual experiments. Results are represented as mean ± SEM. Scale bar indicates 200 μm in B.

**Figure S2.*miR-669a-5p* inhibitor does not suppress adipogenic differentiation of C3H10T1/2 cells**

C3H10T1/2 cells were transfected with inhibitor control or inhibitor *miR-669a-5p* (200 nM) on day 0 and day 3 after adipogenic differentiation, the cells were collected on day 6 for analysis.

(A-B) Lipid accumulation was assessed by Oil Red O staining, and the absorbance was measured at 510 nm wave length. A representative image of three independent experiments is shown in A.

(C)Western blot to evaluate the protein levels of brown fat genes PPARγ, PGC-1α and UCP1, β-ACTIN was used as a loading control. n = 3 per group.

(D) Quantitative densitometry of the western blots showed in C.

Data are representative of at least three individual experiments. Results are represented as mean ± SEM. Scale bar indicates 200 μm in A.

**Figure S3. Gene Ontology (GO) enrichment analysis during differentiation of 3T3-L1 cells treated by mimic *miR-669a-5p* or mimic control**

**Figure S4.The expression of *miR-669a-5p* and *Sfbmt2* in adipose tissue from obese mice**

8-week-old male C57BL6/J mice were fed with a standard chow diet or high fat diet for 8 weeks.

(A) The body weight and body fat ratio of mice fed with chow or high fat diet (HFD). n = 5 per group.

(B) RT–qPCR analysis for *miR-669a-5p* expression in eWAT and BAT of mice fed with chow or HFD, normalized to *U6* expression. n = 5 per group.

(C) RT–qPCR analysis for *Sfmbt2* expression in eWAT and BAT of mice fed with chow or HFD, normalized to *β-actin* expression. n = 5 per group.

Data are representative of at least three individual experiments. Results are represented as ± SEM. ***p* < 0.01 versus chow diet group.

**Figure S5. The expression profiles of *Sfmbt2* and** **some randomly selected C2MC members during the adipogenic differentiation of 3T3-L1 cells**

(A) RT–qPCR to analyze the expression of *Sfmbt2* in 3T3-L1 cells during differentiation, normalized to *β-actin* expression. n = 3 per group.

(B-C) RT–qPCR to analyze the expression levels of some C2MC members (*miR-297a-5p* and *miR-467a-5p*) in 3T3-L1 cells during differentiation, normalized to *U6* expression. n = 3 per group.

Data are representative of at least three individual experiments. Results are represented as mean ± SEM. **p* < 0.05, ***p* < 0.01, ****p* < 0.001 versus day 0 group.

**Figure S6.** **The expression profiles of *Sfmbt2* and some randomly selected C2MC members during the adipogenic differentiation of C3H10T1/2 cells**

1. RT–qPCR to analyze the expression of *Sfmbt2* in C3H10T1/2 cells during adipogenic differentiation, normalized to *β-actin* expression. n = 3 per group.

(B-D) RT–qPCR to analyze the expression levels of some C2MC members (*miR-297a-5p and miR-467a-5p*) in C3H10T1/2 cells during adipogenic differentiation, normalized to *U6* expression. n = 3 per group.

Data are representative of at least three individual experiments. Results are represented as mean ± SEM. **p* < 0.05, ***p* < 0.01, ****p* < 0.001 versus day 0 group.
