# Supplementary material for: miR-669a-5p promotes adipogenic differentiation and induces browning in preadipocytes
Source: Adipocyte. 2022 Jan 30;11(1):120–32. doi: 10.1080/21623945.2022.2030570 (PMC8803067; doi:10.1080/21623945.2022.2030570)
Supplement: Supplemental Material [file KADI_A_2030570_SM9851.zip › supplementary/Table S1.docx]

**Table S1. List of gene specific primers used for RT-qPCR**

| Gene symbol | Forward Sequence (5' to 3') | Reverse Sequence (5' to 3') |
| --- | --- | --- |
| *Acadl* | TCTTTTCCTCGGAGCATGACA | GACCTCTCTACTCACTTCTCCAG |
| *Acadm* | AGGGTTTAGTTTTGAGTTGACGG | CCCCGCTTTTGTCATATTCCG |
| *Acadvl* | CTACTGTGCTTCAGGGACAAC | CAAAGGACTTCGATTCTGCCC |
| *Aco2* | ATCGAGCGGGGAAAGACATAC | TGATGGTACAGCCACCTTAGG |
| *Actb* | GGCTGTATTCCCCTCCATCG | CCAGTTGGTAACAATGCCATGT |
| *Atp5a1* | TCTCCATGCCTCTAACACTCG | CCAGGTCAACAGACGTGTCAG |
| *Bmper* | GAGCTCTGTCAAGGCACAGT | ACAGGACCGGTAGAAAGTGG |
| *Cpt1b* | GCACACCAGGCAGTAGCTTT | CAGGAGTTGATTCCAGACAGGTA |
| *Cpt2* | CAGCACAGCATCGTACCCA | TCCCAATGCCGTTCTCAAAAT |
| *Crat* | GCTGCCAGAACCGTGGTAAA | CCTTGAGGTAATAGTCCAGGGA |
| *Cs* | GGACAATTTTCCAACCAATCTGC | TCGGTTCATTCCCTCTGCATA |
| *Fabp4* | AACGAGATGGTGACAAGCTG | AATTTCCATCCAGGCCTCTT |
| *Hadha* | TGCATTTGCCGCAGCTTTAC | GTTGGCCCAGATTTCGTTCA |
| *Hadhb* | ACTACATCAAAATGGGCTCTCAG | AGCAGAAATGGAATGCGGACC |
| *Idh3a* | TGGGTGTCCAAGGTCTCTC | CTCCCACTGAATAGGTGCTTTG |
| *Lpl* | GGATGGACGGTAAGAGTGATTC | ATCCAAGGGTAGCAGACAGGT |
| *Mdh2* | TTGGGCAACCCCTTTCACTC | GCCTTTCACATTTGCTCTGGTC |
| mt-*Nd1* | CCCATTCGCGTTATTCTT | AAGTTGATCGTAACGGAAGC |
| *Ndufb8* | TGTTGCCGGGGTCATATCCTA | AGCAT CGGGTAGTCGCCATA |
| *Nrf1* | GGTGTTTGGCGCAGCACCTT | TCTGGGATAAATGCCCGAAGCT |
| *Pgc1a* | AATGCAGCGGTCTTAGCACT | GTGTGAGGAGGGTCATCGTT |
| *Ppargc1b* | TCCTGTAAAAGCCCGGAGTAT | GCTCTGGTAGGGGCAGTGA |
| *PPARγ* | AAGAGCTGACCCAATGGTTG | ACCCTTGCATCCTTCACAAG |
| *Prdm16* | CAGCACGGTGAAGCCATTC | GCGTGCATCCGCTTGTG |
| *Perilipin2* | GACCTTGTGTCCTCCGCTTAT | CAACCGCAATTTGTGGCTC |
| *Rgs4* | TGAAACATCGGCTGGGGTTC | GGCTTACCCTCTGGCAAGTT |
| *Sdhb* | CTGAATAAGTGCGGACCTATGG | AGTATTGCCTCCGTTGATGTTC |
| *Sdhc* | GCTGCGTTCTTGCTGAGACA | ATCTCCTCCTTAGCTGTGGTT |
| *Sdhd* | TGGTCAGACCCGCTTATGTG | GGTCCAGTGGAGAGATGCAG |
| *Sh3d21* | CCCGGAACTCTGGCTTCAAT | ATGCAGAGTGCTGTTGGGAA |
| *Tfam* | GCAAAGGATGATTCGGCTCAGGGAA | CCGGATCGTTTCACACTTCGACGG |
| *Ucp1* | GGCCTCTACGACTCAGTCCA | TAAGCCGGCTGAGATCTTGT |
| *Uqcrc2* | AAAGTTGCCCCGAAGGTTAAA | GAGCATAGTTTTCCAGAGAAGCA |
| *miR-669a-5p* | AGTTGTGTGTGCATGTTCATGTCT |  |
| *miR-U6* | CGCAAGGATGACACGCAAATTC |  |
| *miR-297a-5p* | ATGTATGTGTGCATGTGCATGT |  |
| *miR-467a-5p* | TAAGTGCCTGCATGTATATGCG |  |
