# Supplementary figures and images for: miR-669a-5p promotes adipogenic differentiation and induces browning in preadipocytes
Source: Adipocyte. 2022 Jan 30;11(1):120–32. doi: 10.1080/21623945.2022.2030570 (PMC8803067; doi:10.1080/21623945.2022.2030570)

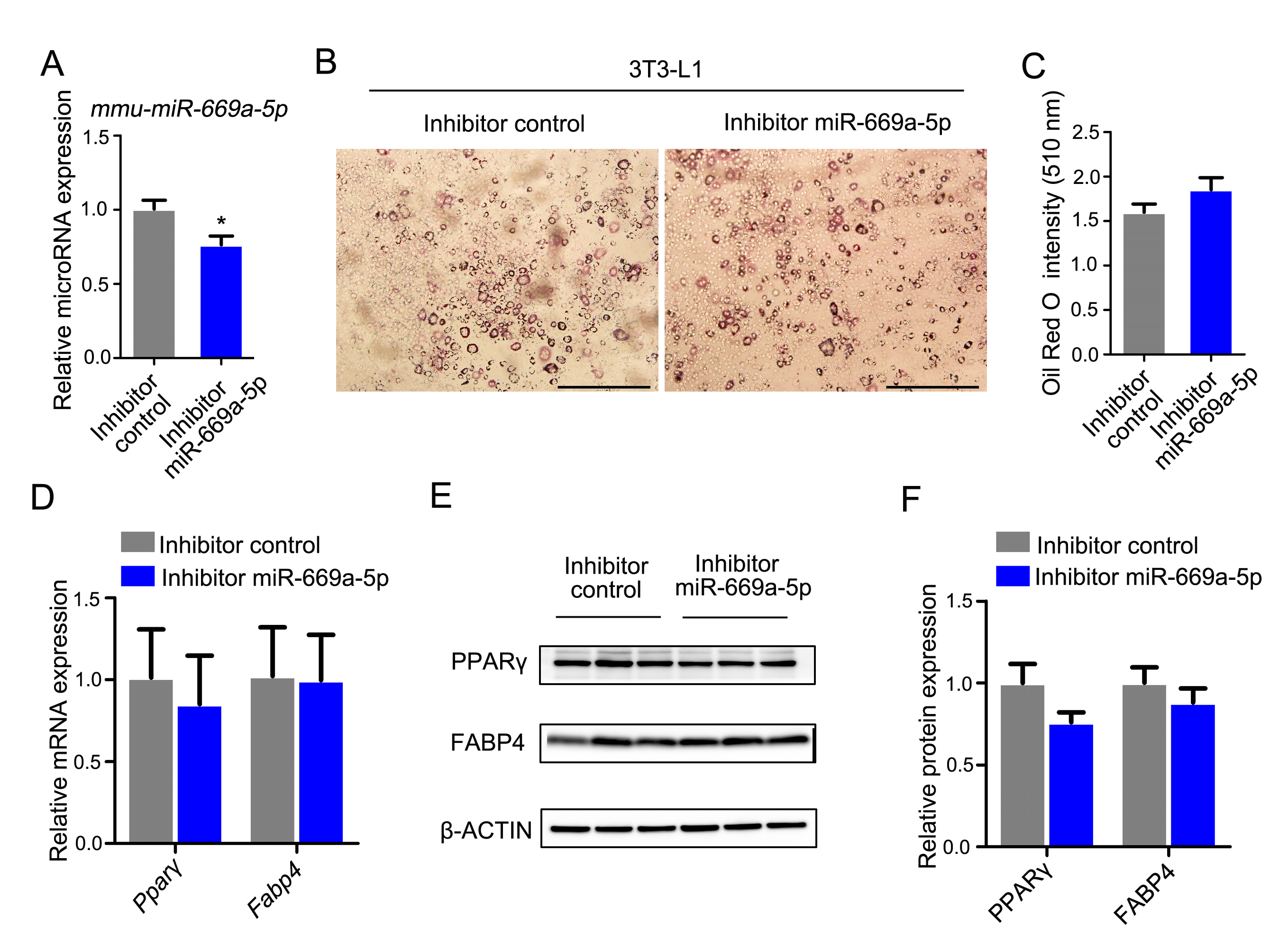

Supplement: Supplemental Material [file KADI_A_2030570_SM9851.zip › supplementary/Figure S1.tif]

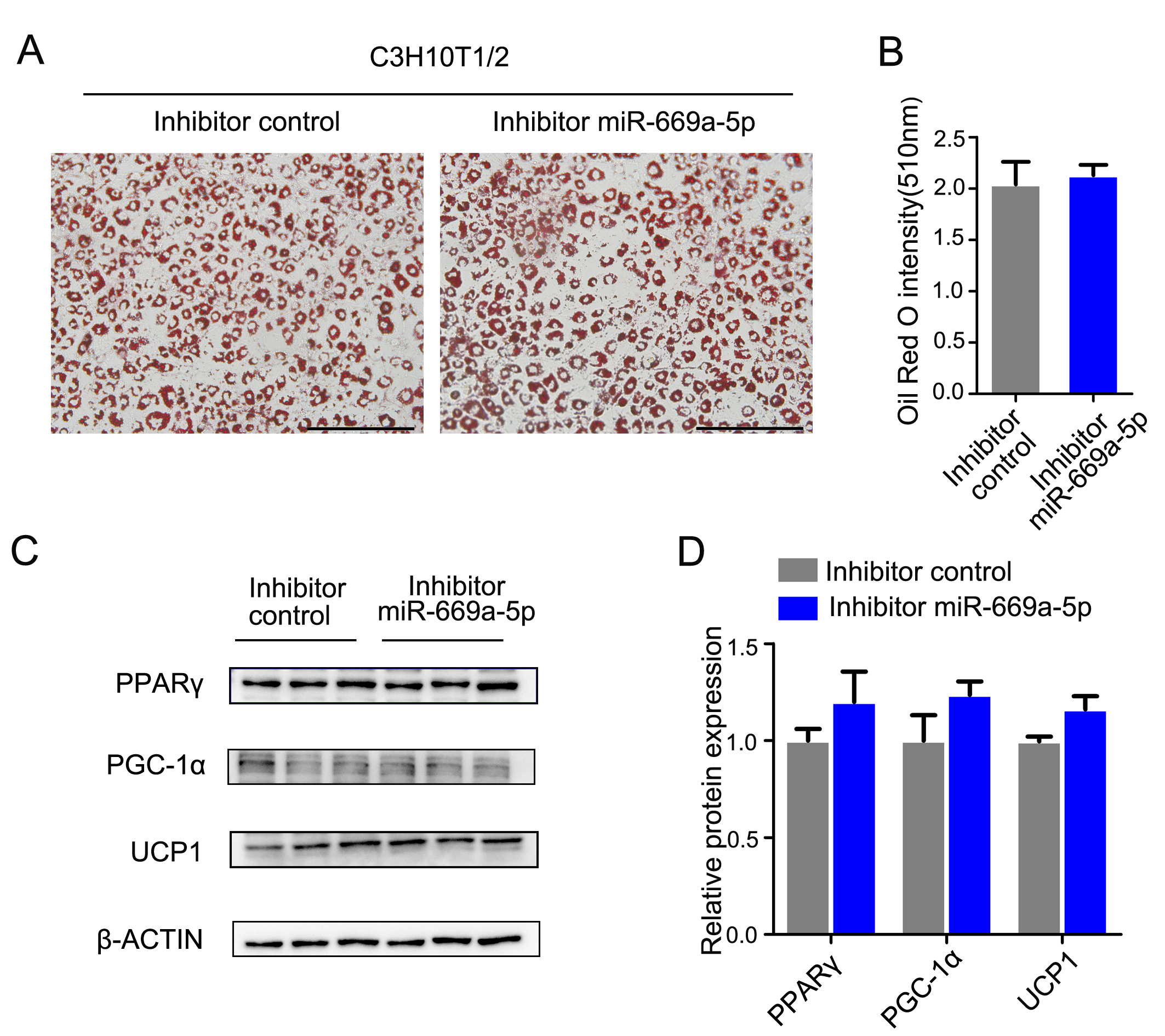

Supplement: Supplemental Material [file KADI_A_2030570_SM9851.zip › supplementary/Figure S2.tif]

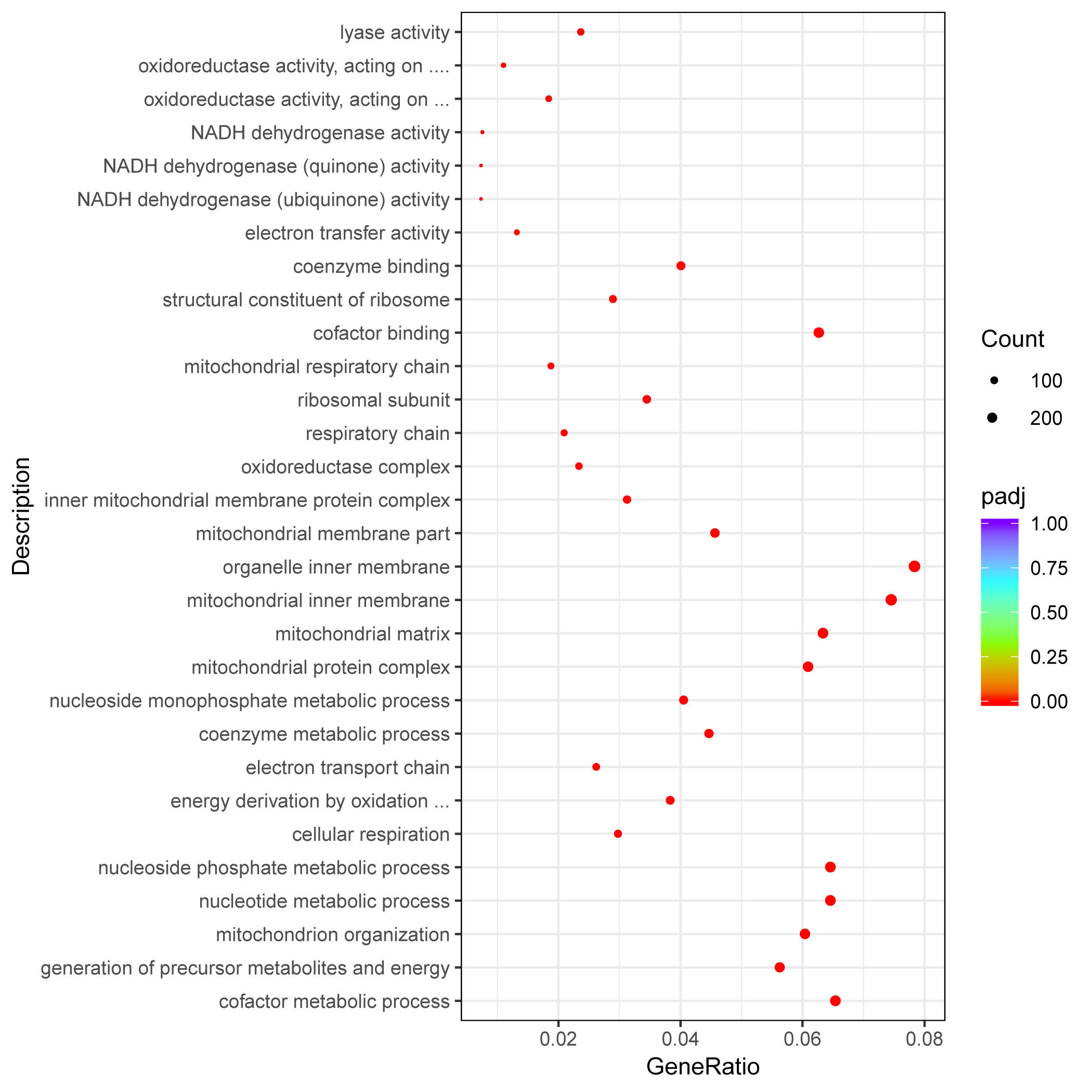

Supplement: Supplemental Material [file KADI_A_2030570_SM9851.zip › supplementary/Figure S3.tif]

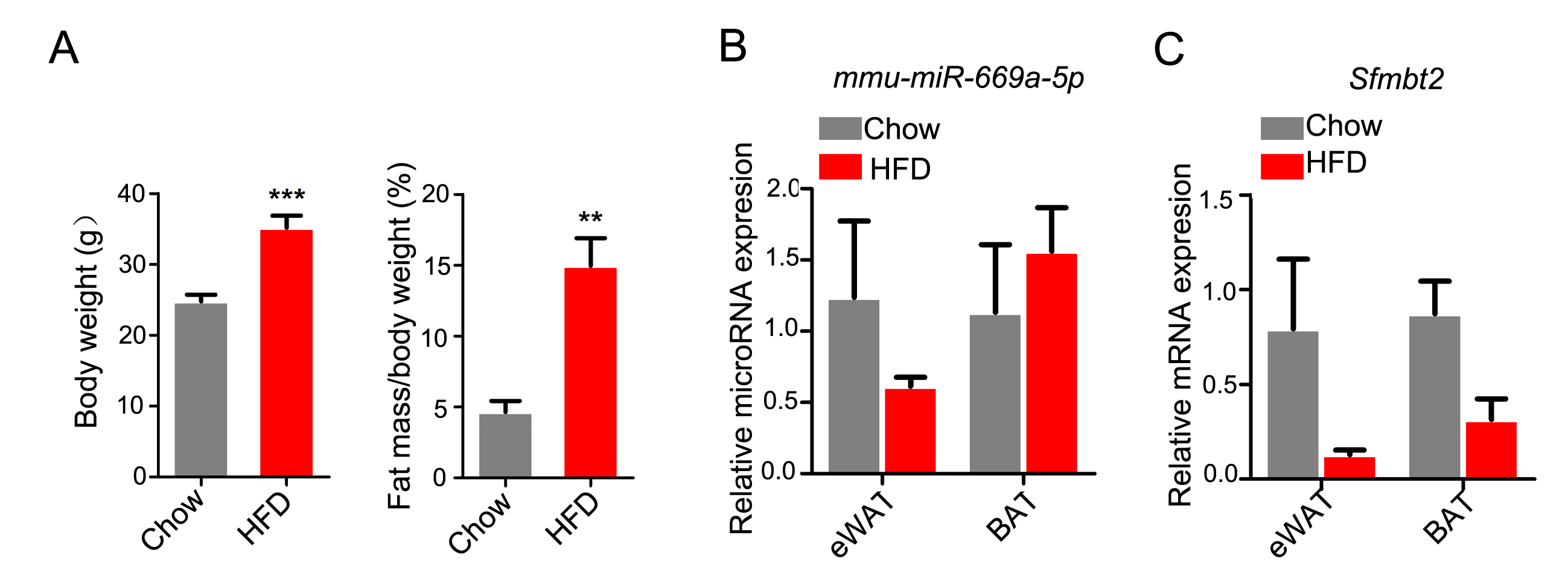

Supplement: Supplemental Material [file KADI_A_2030570_SM9851.zip › supplementary/Figure S4.tif]

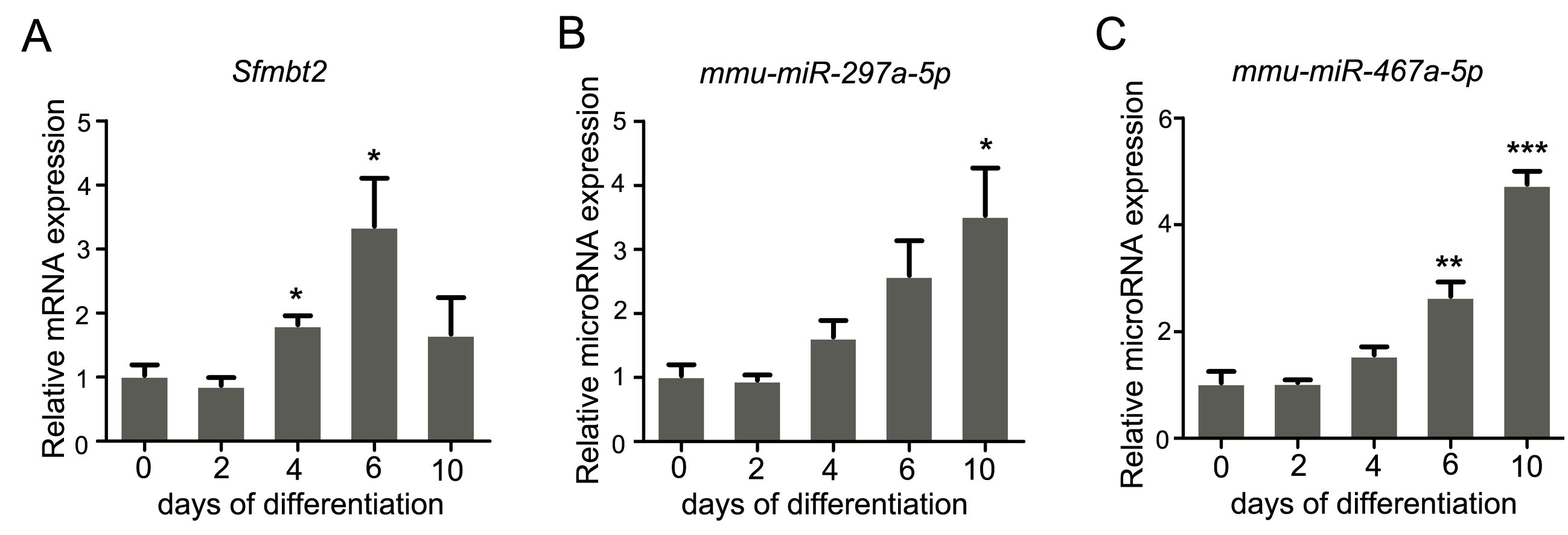

Supplement: Supplemental Material [file KADI_A_2030570_SM9851.zip › supplementary/Figure S5.tif]

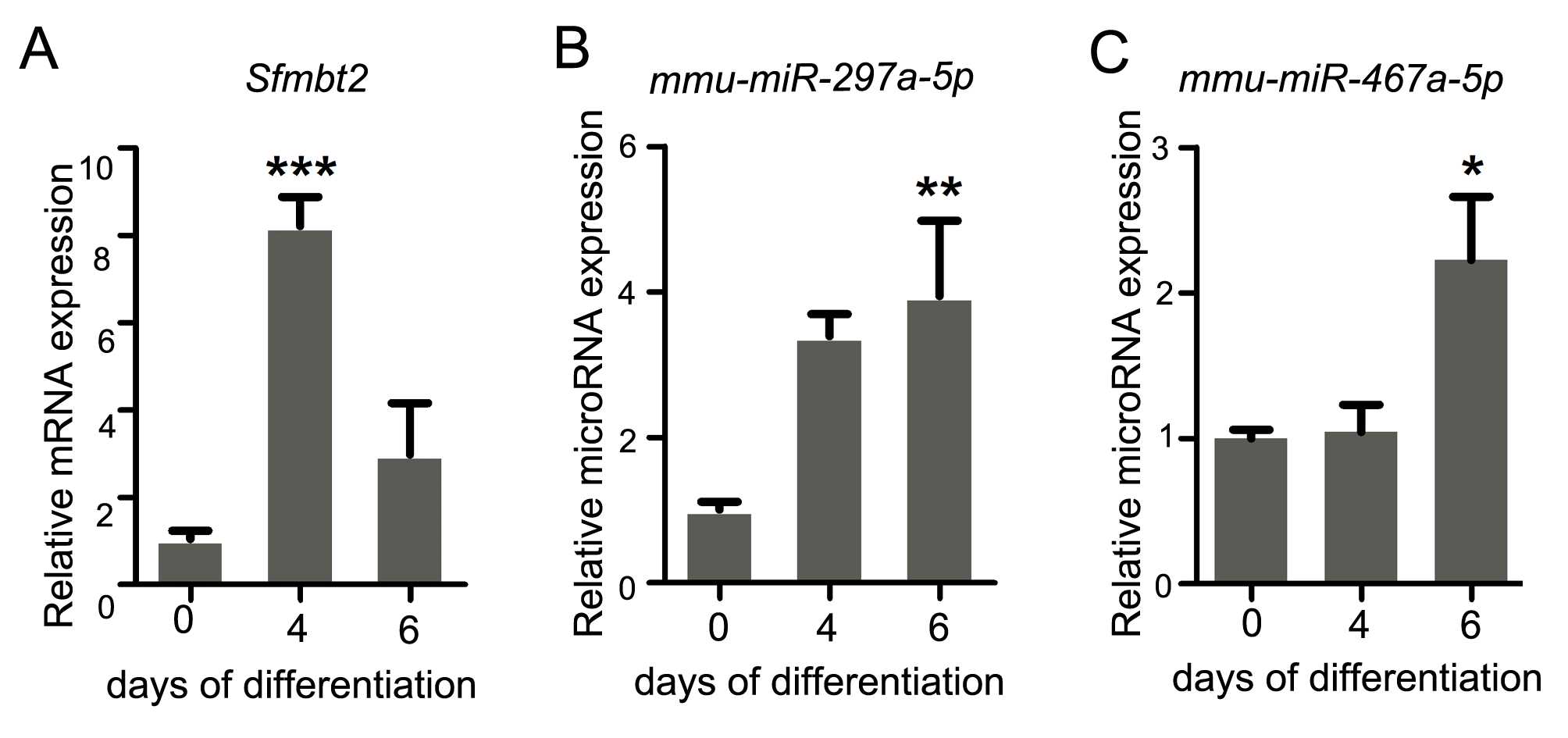

Supplement: Supplemental Material [file KADI_A_2030570_SM9851.zip › supplementary/Figure S6.tif]
